# Living with haemodialysis in Sri Lanka: a qualitative study on patient adaptation and care perceptions

**DOI:** 10.1186/s12882-025-04728-6

**Published:** 2026-01-06

**Authors:** Chalani Lasanthika, Kamani Wanigasuriya, Usha Hettiaratchi, Thamara Dilhani Amarasekara, Christine Sampatha Evangeline Goonewardena

**Affiliations:** 1 Department of Nursing and Midwifery, Faculty of Allied Health Sciences, University of Sri Jayewardenepura, Gangodawila, Nugegoda, Sri Lanka; 2 Center for Kidney Research, Faculty of Medical Sciences, University of Sri Jayewardenepura, Gangodawila, Nugegoda, Sri Lanka; 3 Department of Biochemistry, Faculty of Medical Sciences, University of Sri Jayewardenepura, Gangodawila, Nugegoda, Sri Lanka; 4 Department of Community Medicine, Faculty of Medical Sciences, University of Sri Jayewardenepura, Gangodawila, Nugegoda, Sri Lanka; 5 Non Communicable Diseases Research Centre, University of Sri Jayewardenepura, Gangodawila, Nugegoda, Sri Lanka

**Keywords:** Chronic kidney failure, Hospital haemodialysis units, Qualitative research, Renal dialysis, Sri Lanka

## Abstract

**Background:**

The increasing prevalence of individuals undergoing haemodialysis (HD) compels the necessity of person-centred care to minimise treatment-related complications and improve the quality of life. Although the person-centred approach has been widely discussed, it remains underutilised in HD care, particularly in low- to middle-income countries. Understanding how patients perceive their care is crucial for developing tailored interventions and achieving desirable health outcomes. Therefore, this study was aimed to explore patients’ experiences of HD care in three selected government hospital dialysis units in Colombo District, Sri Lanka.

**Methods:**

One-to-one, semi-structured interviews were conducted with a purposive sample of twelve participants undergoing HD for more than six consecutive months and receiving at least one dialysis session per week. Interviews were conducted from April to December 2022 until data saturation was reached. All interviews were digitally recorded, transcribed verbatim and analysed using the qualitative content analysis proposed by Graneheim and Lundman.

**Results:**

An overarching theme, ‘Adapting to a Disrupted Life Trajectory’ with four categories emerged: Burden of living with HD, Physical and psychological consequences of treatment, Coping and adjustment mechanisms, Healthcare system and service experiences. Daily life was often a burden for the participants due to physical and psychological challenges, numerous life restrictions and self-care burden. Despite these altered life routines, patients adapted coping mechanisms to regain a sense of control and maintain continuity of their treatment. Stigma associated with the terminal illness, famiy dynamics, and internal coping through spirituality shaped unique patient adaptation to the illness experience. Participants expressed a need for accessible HD care that is high in quality, safe, and delivered with compassion.

**Conclusion:**

A person-centred approach, including goals of care conversations, would help to address patients’ concerns related to the multifaceted burden of HD treatment. Strengthening a well-coordinated, multidisciplinary team including nephrology, palliative care, and community-based services is essential to meet patients’ complex needs and provide specialist support across physical, psychological, social, and spiritual domains during the illness trajectory in a more holistic and timely manner.

**Supplementary Information:**

The online version contains supplementary material available at 10.1186/s12882-025-04728-6.

## Background

Chronic kidney disease (CKD) is a serious public health concern, prevalent among approximately 9.5% of the global population [1], particularly placing a significant burden on developing countries [2]. The patients with CKD in the most advanced stage, referred to as ‘kidney failure’, are treated with renal replacement therapy (RRT), which includes dialysis and kidney transplantation. The estimated global prevalence of all forms of maintenance dialysis was 823 per million population [3]. Of this population, approximately 89% accounted for haemodialysis (HD) due to its accessibility in most countries [4]. HD is the major RRT across many countries, utilising a considerable proportion of national healthcare expenditure [1, 5].

The number of patients on dialysis continues to grow rapidly in low- to middle-income countries, driven by increased access to dialysis, population ageing, rising rates of hypertension and diabetes mellitus, and exposure to certain toxic environmental factors [6]. Over the past twenty years, the burden of CKD in Sri Lanka has grown significantly, mainly because of the increasing prevalence of CKD of Unknown aetiology (CKDu), especially in the North Central Province [7, 8], alongside other contributing factors. Consequently, an increasing number of patients reach the terminal stage of CKD and require RRT to survive. For most of these patients, HD is the primary treatment option [7], as kidney transplantation is not a viable option due to a lack of donors or is limited by several early and late complications, including high infection rates following deceased donor transplants. Consequently, an estimated 4.8% patients in Sri Lanka found to return to dialysis following unsuccessful transplantation [9], contributing to further demand on HD services.

Although HD offers significant survival benefits to certain individuals [10], it has not been proven to be beneficial for all, particularly those in advanced age and with multiple comorbidities [11]. The chronic and demanding nature of HD imposes a significant burden on the physical, psychological, social, emotional and economic aspects of the lives of individuals, and as a result, they may have to adapt to drastic changes in their lifestyle [12–14]. HD treatment can deteriorate the functional status of patients over time, often accompanied by multiple physical and psychological symptoms [5, 15] that disrupt their day-to-day functioning, including fatigue, fluctuating blood pressure, dizziness, shortness of breath, muscle cramps, oedema, pain, sleep disturbances, anxiety and depression [5, 16]. In addition, these patients continued to experience a wide range of emotional and cognitive challenges, including fear and uncertainty about future care and impending death, feelings of grief, and a gradual decline in memory over time [5]. These distressing symptoms and psychological burden have a profound impact on the quality of life of patients receiving HD [16].

A recent review has identified several key areas of experience of patients receiving HD, including loss of independence, disruptions to daily routines, the importance of relationships with healthcare professionals, the need for self-management, the role of family support, impaired social function, and emotional coping [14]. In addition, it highlights numerous unmet supportive care needs among patients on HD, including practical needs (finances, employment, transport issues), spiritual and existential concerns, sexual and intimacy needs and needs of service and care improvements [14]. These unmet needs and the devastating nature of the consequences associated with the disease and HD treatment require careful assessment and monitoring of the patient’s condition alongside planning and implementing more person-centred, comprehensive care to minimise potential complications and improve quality of life [12].

Safe and person-centred care is a key characteristic of quality healthcare, which contributes to the desired health outcomes of individuals [17]. Understanding patient care needs and preferences is vital for planning and delivering tailored, high-quality care [18]. Conversely, studying patient satisfaction offers valuable insights into areas of care that need improvement [19] and reflects expectations of patients whether they met the desired health outcomes from the healthcare that they received [20].

The patient preference of outcomes of HD care includes the satisfaction with care, ensuring the safety of the treatment and improving the quality of life while coping with drastic changes and burdens in their everyday life [21]. Nevertheless, these outcomes may not be uniform for every individual, as they experience unique illness trajectories [16] under various circumstances of care, including different contexts, income levels and supportive services. People in low-resource settings experience formidable constraints in accessing and continuing HD treatment, leading to poor outcomes [22] and experiencing a double burden of care to both patients and their family caregivers [23]. Although the literature suggests how Sri Lankan family caregivers experience exhaustion and burdens of HD treatment [24], patients experience related to their own care is lacking in the literature.

Further, Sri Lanka has its unique challenges in health care as it offers free of charge HD treatments from a limited number of state-funded dialysis units for patients with different etiologies and coming from different areas of the country. Therefore, these units are overcrowded with patients who require chronic dialysis, resulting in fewer and prioritised patients to be dialysed approximately one or two days per week [25]. Although there are a limited number of private sector hospitals providing HD, those are not affordable for many people due to the high cost [7, 25]. Despite recommended dialysis schedule, some patients attend only one session to save money, as long-distance travel makes treatment physically and financially challenging, leading to complications and early mortality [7].

The present study was conducted in major dialysis units in Colombo District, Sri Lanka, where patients with diverse clinical profiles, including those referred from other regions, including rural areas, receive HD care. Regardless of the aetiology, patients receiving HD in dialysis units in Sri Lanka face similar issues within their healthcare context, including resource constraints such as limited nephrology specialists, inadequately trained personnel to deliver care and limited healthcare infrastructure [25].

Given the increasing number of patients undergoing HD and the limited access to adequate HD services in Sri Lanka, a thorough understanding of patients’ experiences with their care is vital for designing context-specific, realistic, patient-centred interventions that reduce the disease and treatment burdens on patients, their families, and the healthcare system. There remains a significant gap in evidence specifically concerning how patients perceive their own care and adapt their lives in response to the burden of kidney disease and HD treatment, especially within the South Asian context, including Sri Lanka, highlighting a need for evidence to better understand this context-specific phenomenon.

## Methods

### Aim

The study was aimed to explore patients’ experiences of HD care in three selected government hospital dialysis units in Sri Lanka.

### Study design

A descriptive qualitative study with an exploratory design and an inductive approach [26] was conducted to comprehensively describe experiences of patients related to HD treatment and care. The study was reported based on the Standards for Reporting Qualitative Research (SRQR) guidelines checklist (Supplementary file 1).

### Study setting and participants

A purposive maximum variation sampling [27, 28] (variation by age, gender, educational level, income, duration of the kidney disease and receiving HD) was used to recruit a diverse group of participants for the interviews from the dialysis units of Colombo South Teaching Hospital, National Institute for Nephrology Dialysis and Transplantation and National Hospital of Sri Lanka, Colombo. These are major dialysis units in the capital city of Sri Lanka, which cater for patients from different ethnic groups and social classes and are admitted from various places in the country. The inclusion criteria for participants were: Patients who received HD treatment for more than six consecutive months according to a routine schedule for at least one session per week, those who were aged above 18 years and able to understand or speak Sinhala or English language from all ethnic groups. Exclusion criteria were: Patients who were on peritoneal dialysis (PD) and were critically ill or with mental disabilities due to the inability to acquire reliable information.

The average sample size for a descriptive exploratory design is 15 participants. However, the design is flexible with regard to the sample size, and the researcher has the responsibility to justify the sample size if there is a strong rationale for selection and if it is sufficient to achieve the aims of the study [28, 29]. Therefore, the sampling process was conducted until no new information was found. A total of twelve participants were interviewed.

### Data collection

One-to-one, semi-structured interviews were carried out from April to December 2022 using an interview guide (Supplementary file 2) to capture a wide range of perspectives related to the research topic and for the most effective use of limited resources [30]. The first author (CL), who was trained in conducting qualitative interviewing, conducted face-to-face semi-structured interviews in a well-ventilated, empty room within each dialysis unit, where the privacy and confidentiality of the participants were fully ensured.

An interview guide was prepared by the first author (CL) based on an intensive literature review [12, 31, 32] and further refined by obtaining the opinions of qualitative research experts (university academics) and subject experts (dialysis nurses). Two pilot interviews were conducted with patients undergoing HD to check the clarity and comprehensibility of the questions in the interview guide, potential barriers to achieving desired responses from the participants, and to determine the duration of time needed for an interview. The interviews were carried out by the first author (CL) after obtaining written informed consent from the participants until the data saturation point, meaning that no new information was obtained from subsequent interviews. Interviews were carried out without disturbing the dialysis treatment and the freedom of the other patients, and also without interfering with the dialysis unit routines. Shortly after an interview, the impressions on the interview were noted, which comprised both observations and interpretations to assist in remembering and reducing the loss of data when performing data analysis [30]. All the interviews were audio-recorded and later transcribed verbatim by the first author (CL). Each interview lasted between 18 and 49 min. All the participants filled out a form with socio-demographic details before the interview. The description of the participants is given in Table 1.

**Table 1:** Participants’ characteristics (*n* = 12)

Variable	Category	*n*	%
Age	< 35 years	3	25.0
	36–50 years	4	33.3
	> 50 years	5	41.7
Gender	Female	7	58.3
	Male	5	41.7
Marital status	Married	10	83.4
	Unmarried	1	8.3
	Widowed/divorced	1	8.3
Race	Sinhala	11	91.7
	Tamil	1	8.3
Religion	Buddhism	10	83.4
	Christian/Catholic	1	8.3
	Hindu	1	8.3
Family type	Nuclear	9	75.0
	Extended	3	25.0
Residence	Own home	10	83.4
	Rented house	2	16.7
Educational level	Not attended school	1	8.3
	Ordinary level (up to grade eleven)	5	41.7
	Advanced level (up to grade thirteen)	3	25.0
	University education	3	25.0
Employment status	Yes	3	25.0
	No	9	75.0
Duration of having chronic kidney disease	1–5 years	10	83.3
	More than 5 years	2	16.7
Haemodialysis duration	Less than 1 year	2	16.7
	1–5 years	10	83.3
Haemodialysis sessions per person/week	Only one day	2	16.7
	Two days	9	75.0
	Three days	1	8.3

### Preunderstanding

Two authors (CL and TA) have a background as registered nurses, and have expertise in treating and caring the patients with chronic kidney disease receiving HD. All authors have experience as researchers in the clinical field, and two authors have experience as researchers in the qualitative field.

### Data analysis

Qualitative content analysis by Granehiem and Lundman (2004) was utilised. In content analysis, the findings are presented as categories and/or themes [33]. In the present study, only manifest content analysis (patterns that are visible in the text) was performed, and the latent content was not considered in interpretation. During the process of content analysis, the following steps were involved; Reading the transcribed narrative text several times to get an overall understanding, extracting the meaning units in the text relating to the research question (description close to the text), condensing the meaning units (interpreted with underlying meaning), labelling the meaning units with codes (coding) and in the last step, similar codes were grouped into sub-categories and then categories into themes [34]. All sub-categories, categories and the overarching theme were evaluated by each member of the supervisory team separately. The findings of the study were presented using quotes from the participants for each sub-category.

### Trustworthiness

The framework proposed by Lincoln and Guba was followed to maintain the trustworthiness of the qualitative study in terms of credibility, dependability, transferability, and confirmability [35]. Credibility was maintained by the first author through appropriate communication with participants, prolonged engagement, and dedicating sufficient time for data collection to gain an in-depth understanding. Additionally, continuous discussions with supervisors during the content analysis of interviews helped improve the trustworthiness of the findings. For dependability, the study kept detailed documentation, used participants’ comments, and decoded texts to allow readers to verify the findings [36]. Transferability, which refers to the extent to which findings can be applied to other settings or groups [37], was ensured by employing maximum variation purposive sampling. Confirmability was achieved through data triangulation, using in-depth individual interviews and observations made during the interviews, as well as referring to previous research findings [35].

### Ethical approval

The study involved human participants and was conducted in accordance with the Declaration of Helsinki [38]. The study was approved by the Research Ethics Committees of the Faculty of Medical Sciences, University of Sri Jayewardenepura (Application No.06/19), The National Hospital of Sri Lanka (AAJ/ETH/COM/2021/JULY) and Colombo South Teaching Hospital (Application No.994). Written informed consent was obtained from all the participants before taking part in the study. The participants were informed about audio recording of the interviews. The participation was voluntary, and participants had the right to withdraw their consent from the study at any time and without explanation. They were also assured that whether they participated or not would not affect the care or treatments that they received. Participants were informed that their personal data would remain confidential. Pseudonyms were used when transcribing the interviews to prevent disclosure of the identities of the participants. All non-electronic items (transcripts with real names and data) were kept locked in and key and all electronic data were kept password protected. Only the first author (CL) has access to the original data.

## Results

An overarching theme, ‘Adapting to a Disrupted Life Trajectory’, emerged from the analysis along with four categories. For the schematic presentation of categories and subcategories, see Table 2.

**Table 2:** Themes, categories and subcategories related to patients’ experiences of haemodialysis care

Theme	Categories	Sub-categories
‘Adapting to a Disrupted Life Trajectory’	Burden of living with Haemodialysis	**•** Restrictions on the daily routine **•** Time-consuming care **•** Financial burden **•** Self-perceived burden
	Physical and Psychological consequences of treatment	**•** Distressing experience with haemodialysis initiation **•** Reduced self-esteem related to bodily decline and illness-related stigma **•** Living with uncertainty
	Coping and Adjustment Mechanisms	**•** Accepting the reality **•** Looking at the situation positively **•** Believing in religion and karma **•** Satisfactory adjustment to daily living **•** Active participation with own care **•** Peer/social support **•** Family involvement with care **•** Feeling of being persuaded and empowered by the health care team.
	Healthcare System and Service Experience	**•** Patients’ expectations for safe and compassionate care **•** Perceived quality of haemodialysis **•** Challenges in accessing haemodialysis services **•** Poor coordination of care

### Category 1: burden of living with HD

HD imposed numerous behavioural and social restrictions on the participants, disrupting their personal and daily routine. The participants’ notions reflected that the burden can arise not only from external sources but also from how they perceived or responded to those circumstances internally. One participant described the sense of disruption, loss of daily routine and the difficulty in adapting to the changed life circumstances due to HD treatment.

*There was a way of spending my daily life. I used to have a busy and active work life before. So*,* It’s really hard to face this difference* (Participant J)

Another participant described the economic and personal life impact because of early retirement from the job to meet the demands of HD treatment.

*I lost my job because of this (haemodialysis). Otherwise*,* I would have been able to work for another eight years. But I had to quit my job earlier* (Participant A)

Apart from the external burden, one participant expressed deep emotional distress and a feeling of being a burden to the family, both financially and emotionally.

*I am suffering from this illness. It would be better if I died at once than suffering like this. This is a huge burden for my children and husband. Once I die*,* at least other people will live without any problem. See how much money has been spent on these treatments. I have wasted their money.* (Participant F)

Participants expressed the emotional and physical struggle they faced because of the several life disruptions and sacrifices they had to make due to the HD treatment. One participant expressed that she could no longer engage in her previous responsibilities as fully as she once did.

*I can’t spend a lot of time on household work because of the time spent on dialysis treatment and travelling to the dialysis unit. I am a mother who was always behind my kids. But now*,* sometimes during the periods that I stay in the hospital*,* there is no one to look after them.* (Participant E)

### Category 2: physical and psychological consequences of treatment

Participants described having distressing experiences when the HD was initiated and subsequently reported experiencing a physical decline, such as lack of energy, changes in body image and fatigue. A feeling of being dependent and disabled was described by many of them. One participant described how fatigue and loss of physical functioning led to a sense of psychological distress.

*Dialysis started in August. Then*,* it changed my whole life; day-to-day*,* everything changed. I can’t cook*,* I can’t wash my clothes*,* I feel tired even if I walk 2 feet. When this happened*,* I felt like my head had been hit by a heavy stick.* (Participant J)

The participants described how they feel embarrassed because of body image changes, reflecting how they respond emotionally to the physical side effects.

*After dialysis*,* my weight decreased by 15–20 kg. Now the hair is falling*,* the skin is dry. It’s a bit embarrassing when I see these changes in my body. When these happened*,* it was like a shock to me* (Participant K)

Some participants described the reluctance to reveal their situation to other people in society because of the stigma associated with end-of-life disease. As a result, they had refrained from social participation and relationships.

*I have many friends. But*,* no one was told about my disease. No one knows that I am a patient*,* only my best friend knows. How could I tell them? That’s why I don’t talk to anyone now* (Participant L*)*

Another participant described living with uncertainty and deep emotional resignation with limited hopes for the future.

*I don’t know how destiny will affect me. but I’ve come to accept whatever happens*,* whenever it happens. I feel like there’s nothing particularly meaningful left in my life now*,* and starting over no longer feels possible. I don’t have much hope for the future. All I know is that I can’t see myself living like this for another three or four years* (Participant C)

### Category 3: coping and adjustment mechanisms

Although HD care was felt as a burden, the concepts of ‘coping’ and ‘support’ were also described as a part of their care trajectory. Participants appeared to encompass a combination of ‘internal’ and ‘external’ mechanisms for coping and adjustment.

Many participants relied on internal coping mechanisms such as accepting the reality of life, looking at the situation positively and believing in religion and karma. As a result, HD became a part of their daily routine and motivated them to adapt their life to a new normal situation.

Participants described their journey from an initial emotional struggle to eventual psychological adjustment, demonstrating adaptation and acceptance of the current situation.

*I’ve gotten used to living with dialysis now. At the beginning*,* I was completely fed up with it*,* but over time*,* I’ve come to accept it. I had to make up my mind. Otherwise*,* continuing with life wouldn’t be possible* (Participant D)

Even though health has changed, participants were determined not to let dialysis define their lives, reflecting inner strength and their ability to hold on to normal life while still doing what they can.

*I haven’t let dialysis interfere with my daily routines. I have no regrets*,* because even though I’m dealing with illness*,* I’m still doing the things in life that matter to me*,* just like I did before* (Participant J)

Some participants accepted that what they experience in the present is not random, that is, because of past actions or the karmic result of their own virtue.

*As in the sermons by Theros*,* all these consequences I experience are a result of past deeds. Everything happens because of the sins and merits that we have committed in the past. Unwholesome actions bring suffering. I believe*,* at least my child cares for me because I have done such merits in my past life* (Participant G)

Participants tend to accept the changes in their lives, realising the impermanence of everything and grounding their sadness with understanding.

*Now my appearance is completely different from before. Sometimes*,* when I look at my old photos*,* I feel really sad. But I remind myself that this is because I am receiving medication for this disease. However*,* when I see other people and their appearance*,* I feel good. Nothing lasts forever* (Participant J)

Many patients articulated that they cannot survive without adhering to the limitations imposed by HD care. Though it is unbearable, one patient stated that he spends a righteous life according to his fate and religion without committing suicide.

*Food and water restrictions became a problem for me. But I know that if I don’t do it*,* I can’t survive. So*,* I have to decide to commit suicide*,* but it’s a stupid decision for me. According to Dharma*,* I am a Buddhist. I know I will go to hell if do like that. So*,* my decision is to spend the time I have in any way.* (Participant B)

Various external adjustment mechanisms such as support systems that patients had appeared to be helpful in navigating their care process. Participants viewed HD care as a shared responsibility among patients, their families, and healthcare staff. While some actively supported the treatment, others showed less involvement or were less attentive to their care.

Some participants had a sense of ownership over their health by actively engaging in HD treatment. Some showed awareness about what they receive as a treatment, understanding their own role in ensuring safe and effective treatment.

*I am always alert while on the dialysis machine. I usually check the fistula to see if there are blood clots*. *Now*,* if you look at other people’s fistula*,* my fistula is 3 years old now. Even*,* last night I took an icepack and massaged it to avoid any distortions. I want to be a little careful because*,* I’m a little shy* (Participant B)

One participant expressed the involvement of family during their HD treatment.

*I have big support from my family means that they (family) give priority to my treatments and care over other household matters* (participant K)

Some participants expressed feeling supported and empowered by healthcare professionals, particularly when experiencing kidney donor rejection.

*The nurses ask about our kidney transplant (KT) plans because we don’t have anyone else to share this with. Even when we talk to family members*,* they often can’t help. But when we share our concerns with the nurses*,* they understand and encourage us to keep searching. During times of donor rejection*,* they support us so we don’t lose hope* (Participant J)

While some have actively participated in treatment adherence, others exhibited neglecting behaviour rationalising the difficulty they endured.

*When it comes to food*,* they say*,* don’t eat that*,* don’t eat this. I don’t know*,* they tell me not to eat the green leaves. There is no such thing as controlling the food. If I control my diet*,* even I won’t be able to walk on the road*,* I will feel dizziness. I don’t feel any difficulty when I eat such foods* (Participant G)

*When I had medicine*,* I felt body aches and can’t work. Therefore*,* I don’t take medicines anymore* (Participant L)

### Category 4: healthcare system and service experience

Participants’ notions about healthcare services often comprised mixed experiences and complex expectations. As HD care was physically and emotionally taxing, participants expected that the healthcare system had to be accessible, reliable and responsive to their needs. Nevertheless, many participants expressed systemic barriers to consistent, safe and coordinated HD care.

One participant described the supportiveness in the dialysis unit under attentive and responsive healthcare staff.

*The dialysis unit is really good*,* the staff identify patients’ problems and is involved in resolving them. They genuinely understand how people experience suffering in this place* (participant I)

Nevertheless, some participants stated the need for a caring and empathetic environment for the patients, ensuring emotional support and dignity, not only providing technical care.

*Any person who comes here for treatment is mentally and physically weak. I think this place must be a better place for that person* (Participant I)

Participants who received treatment at multiple dialysis centres often described noticeable differences in care quality.

*I am currently undergoing dialysis in two hospitals. In doing so*,* I feel that the dialysis procedure is not difficult here. But in the other hospital*,* dialysis treatment is very oppressive from the point of punching* (Participant E)

The psychological readiness and support when commencing the HD treatment were an important aspect of care for the participants. One participant expressed the importance of healthcare support in providing good orientation and psychological preparation to the patients.

*I believe it’s important to gather patients in groups and provide them with clear information about what to expect next*,* such as explaining the dialysis unit and the upcoming stages. This helps them prepare mentally and make informed decisions. Many patients experience intense fear*,* worrying that dialysis means they are near death*,* and some even faint upon seeing these dialysis machines* (Participant B)

Some participants expressed the challenges of accessing HD services, particularly from a different healthcare centre than usual.

*Undergoing dialysis at another hospital is confusing*,* and the staff don’t seem to care. Sometimes*,* I spent the whole day there without an appointment because it’s hard to see other doctors without my regular doctor* (Participant A)

Many participants expressed that they value preventive care than treating the disease. They described priority as the importance of ensuring social awareness on kidney disease, early diagnosis and treatments while giving the second priority for quality and safe HD care. One participant expressed;

*I don’t like dialysis; I would like if we could stop this disease. If we can make people aware about this disease… Actually*,* I was not aware either. Even*,* I’m aware to this level now by accessing information from YouTube and internet after I got this disease.* (Participant J)

## Discussion

This study aimed to explore patients’ experiences of HD care in three selected government hospital dialysis units in Colombo District, Sri Lanka. The overarching theme ‘Adapting to a Disrupted Life Trajectory’ illuminates ongoing adjustments of individuals since the start of the HD treatment, while embracing its difficulties and profound burdens to their daily life. This ongoing process was clearly reflected by the categories: Burden of living with HD, Physical and psychological consequences of treatment, Coping and adjustment mechanisms, and Healthcare system and service experience.

This dynamic process of adaptation of patients undergoing HD clearly aligns with the ‘Chronic Illness Trajectory Framework’ proposed by Corbin & Strauss (1991), reflecting how people live with and manage a chronic illness over time [39]. ‘Chronic Illness Trajectory Framework’ conceptualises chronic illness as a dynamic, ongoing process involving shifting phases of pre-trajectory (occurs before onset of symptoms), trajectory onset (the point where signs and symptoms appear), stable, unstable, acute, crisis (makes significant threat to the physical, social or psychological integrity), comeback, downward progression (deteriorated patient’s recovery), and dying (terminally ill) [39]. The findings of the present study resonate with how participants experienced the disrupted life trajectory through fluctuating phases of chronic illness while grappling with uncertainty and altered life plans. This highlights the importance of holistic, person-centred care as a means to enhance the quality of life of patients, aligning with each phase of the illness trajectory.

A recent systematic review of evidence related to the lived experience of people on maintenance dialysis emerged with key themes of ‘disrupted biographies’ and ‘biographical repair’, which illuminates the findings such as disruption to the normal life with symptoms and functional limitations, uncertainty and psychological distress, self-image and identity changes and coping [16]. The present study reported similar findings in terms of burden and consequences of HD; however, with a different angle reflecting the unique experience of patients being in a low to middle-income country where people find it difficult to access adequate dialysis treatment and relevant care with many constraints, and have a strong cultural influence on their care.

In the present study, the main concerns among patients to feel restricted were behavioural and social confines caused by strict adherence to complex treatment regimens, including medications, dietary and fluid restrictions. Confinement of their life in the home and HD unit made them experience a perceived self (internalised) stigma, following thoughts of ‘life has come to an end’, hopelessness and believing that the society accepts them as incapable and ill. Some have experienced negative comments and unkind attitudes from society about the prognosis of their disease, therefore, they were embarrassing to reveal their disease to others. As a result, many patients left their social relationships and limited their social participation. Similar to the present study findings, existing evidence shows that the people undergoing HD suffered from restrictions in physical performance, employment, sexual and marital life, social participation [40], and behavioural limitations particularly due to diet and fluid restrictions [41–43]. Social stigma on HD was found among Pakistani patients, which was injurious to patient well-being [41]. Currently, there have been a few studies focusing on stigma in patients receiving HD, particularly in the South Asian context, though it can contribute to adverse consequences, with social isolation leading to psychosomatic reactions and impacting quality of life [44].

Commencing HD treatment was often distressing for the patients as it was an unfamiliar experience, which made them anxious and emotionally affected because of the uncomfortable feeling at the initiation of HD and the sudden change to their everyday lives. Some have mentioned their unwillingness to go through HD treatment, although they had to accept it because no other option was available to preserve their life. Nevertheless, many of them expressed that they have gradually adapted to the stressful situation of HD, though it is difficult to tolerate. Similarly, elderly patients with advanced CKD or receiving HD in North Carolina, United States, revealed that patients were shocked when they first heard about their diagnosis, were uncertain about the course of their illness and were unprepared for living with dialysis. Dialysis therapy and adjustments that must be done in their lives were filled with physical, mental and unanticipated challenges [45]. In the present study, the majority of the patients had a fear of being dependent on dialysis and their families, similar to a study in Sweden revealed the experience of feeling vulnerable due to the consequences of the illness and being dependent on their caregivers [46]. Participants in the present study reported having a gradual physical decline following HD, which is consistent with the experience of patients undergoing HD in Ethiopia with body swelling, abdominal distension and darkening of the face [43].

Uncertainty is a major psychological stressor for a patient with a life-threatening disease, which occurs when patients cannot define the meaning of illness-related events [47]. In corresponds with McCormick (2002), the patients of the present study experienced ambiguous symptoms such as trembling in hands and legs, forgetfulness, and trouble sleeping, which they could not explain whether it was due to kidney disease or any other co-morbidity. Also, the unpredictable success of existing treatment options (HD or kidney transplant), change of symptoms over where they feel good and bad in different situations, not having hopes for the future and desire to live, and understanding the reality of life while realising the impermanence of everything described in participants’ notions that meet the attributes of uncertainty. A qualitative study conducted in Iran observed ‘uncertainty’ as the most prominent experience among elderly patients over 60 years undergoing HD [48]. Previous studies conducted among patients undergoing HD in India [49], the United States [50], and Greece [42] also experienced uncertainty and fear about their health, dependency level, and the possibility of a kidney transplant in the future. Advance Care Planning (ACP), which refers to “a process which addresses preferences, values and beliefs of patients related to their current and future care, including end of life preferences” [51], can be used for ongoing planning of appropriate care to alleviate uncertainty and suffering among these patients. Healthcare professionals, particularly Nurses, can address uncertainty of patients by recognising their emotional reactions to it, developing effective communication skills to increase patient satisfaction about treatments, and adopting a multidisciplinary team approach when providing care [52]. However, these supportive care services remain underutilised for patients with kidney failure in Sri Lankan health care system due to a limited number of trained healthcare professionals to provide such care to those who need it most.

Care burden was a significant challenge for the patients to live a normal life. In the present study, patients experienced time-consuming care, financial constraints, and self-perceived burden regarding their own care. Time-consuming care was mainly described due to long waiting hours for HD treatments and time spent for transportation to reach the dialysis centre, which needed to be tackled in a systematic and more organised way by developing sufficient health care resources, including the use of technology for real-time scheduling of appointments, introducing dedicated transport services and expanding access to the satellite dialysis units. Struggling with time-consuming care was also described by the patients receiving HD in several previous studies [40, 41, 53]. The financial burden was the most significant challenge among patients in the present study, similar to the many developing countries such as Pakistan, India and Ethiopia [41, 43, 49] due to the high cost of dialysis treatments in the private sector, transportation, medications, poor financial assistance, and financial commitments to finding a kidney donor for transplantation.

In Sri Lanka, almost all the state-funded dialysis units are overcrowded with an excessive number of patients. Therefore, most of the patients are dialysed only twice per week or less instead of the recommended thrice per week dialysis. Hence, some patients were undergoing additional dialysis treatment from the private sector hospital (to prevent trouble breathing between dialysis treatments), which cost them a lot and led them to substantial financial difficulties [7, 25]. Nevertheless, this is feasible only for patients who can afford their treatment cost; therefore, many patients in Sri Lanka are often left to rely on a limited number of government-funded HD treatments, which are insufficient to manage their symptoms effectively or maintain an adequate level of quality of life.

Therefore, it is crucial to reduce the cost of HD by exploring low-cost treatment approaches, including supportive care approaches and developing a dialysis workforce [25] while effectively utilising available resources to meet the patients’ treatment demands. Integrating routine nephrology care with palliative care, while reinforcing a multidisciplinary approach, particularly involving nurses and social workers across both hospital and community settings, may help reduce the healthcare costs associated with HD and ease the overall burden of the disease on patients and their families. Moreover, it is important to focus on post-dialysis care services to support patient adherence to fluid restrictions, dietary recommendations, and prescribed medications with a tailored care approach [7], particularly in collaboration with both hospital and community care teams. Recent attention has focused on strengthening PD programmes in Sri Lanka, including home-based therapies, exploring lower-cost approaches such as local production of PD solutions in response to the growing number of patients with kidney failure and overcrowding of HD units [25].

This situation may differ from that of patients in countries having high-resourced healthcare systems, where individuals typically receive adequate dialysis (thrice per week), have health insurance to cover treatment costs, higher levels of health literacy, and access to supportive care services. In those settings, patients face a different set of challenges shaped by their context, such as the need for support to maintain independence, transportation and practical assistance, and issues related to parking availability for patients and their relatives when accessing HD units [14].

Patients’ attempts to cope with the life changes imposed by HD treatment were described in the present study, whereas a similar situation was observed in Swedish patients who tried to manage restricted life by recalling what life has offered them in the past [46]. In particular, patients in the present study used different and culturally sensitive strategies to cope with the situation, including accepting reality, looking at the situation positively, and believing in religion and karma. Sri Lankan patients believed in religion and karma to cope with their life changes, particularly spiritual strength to overcome physical and psychological difficulties. Patients were determined to live righteous lives and not give up even when facing troublesome situations. Similarly, the desire to keep living despite challenges was identified from the study conducted in Korea [54]. Yodchai et al. (2016) described the religious and spiritual beliefs of Thai patients about suffering from CKD, including believing CKD is a ‘Karma disease’, making merits to balance their bad karma, mainly emanating within the Buddhist religion and culture [55]. The majority of the patients in the present study were Buddhists, and their primary internal coping mechanism was finding strength from their religious beliefs, particularly through listening to and reflecting on ‘Lord Buddha’s teachings’ (sermons). This spiritual practice helped them to cultivate acceptance of the disease and burden they experience, and continue to value their life despite the hardships they encountered. A recent single-centre study on psychosocial experiences of Sinhala Buddhist patients undergoing HD and their family caregivers in Sri Lanka revealed spirituality and religion as a key coping strategy among patients to relieve physical and mental distress, particularly through cultural practices, such as veneration of the sacred Bodhi tree (worshipping the Bodhi tree), intertwined with Buddhism [56]. Irrespective of the religion, all the patients, who belonged to different religious backgrounds in the present multi-centre study believed their fate and destiny were shaped either by the consequence of karma or God’s will. This finding aligns with the existing literature that indicates spirituality among patients receiving maintenance HD is predominantly expressed through a strong faith in God and believing that the God as being in control of their future [14].

Family-centred care is a unique aspect of care in the Sri Lankan context, and for many patients, family was the primary source of support in navigating the caregiving process. Although caregiving is highly burdensome, the unwavering efforts of family caregivers to hold on to their loved ones and not let them go [24] may have instilled a sense of hope and emotional strength in patients. This strong familial presence likely encouraged patients receiving HD in the Sri Lankan context to endure difficulties and not give up on their lives.

Quality of care is a multi-dimensional construct that cannot be measured on a scale or by analysing its composition [57]. Patient experience is a key indicator of healthcare quality, with patient satisfaction representing a primary outcome of that experience [58]. Although in the present study, patients expressed satisfaction with certain physical and psychological aspects of their care, they also identified several deficiencies in the HD services provided in Sri Lankan hospitals. Due to various challenges in accessing HD, patients expressed a strong need for improved access to quality HD services. Their concerns included limited availability of treatment, lack of flexibility in service delivery at certain hospitals, the high cost of care, and the need for adequately trained healthcare professionals to administer HD in certain healthcare settings. Lack of coordination, dedication, and motivation among certain healthcare staff in providing care was seen by the patients as reasons for certain performance errors and unsafe practices during HD treatment. A similar situation was experienced by patients undergoing HD in Sri Lanka from a recent study, showing a desire for a more engaging approach from medical staff [56]. Patients expressed the need for a more respectful and supportive hospital environment, emphasising the importance of adequate counselling and effective communication. They highlighted the role of such support in promoting treatment adherence, improving nutrition, disease and treatment orientation, ensuring regular follow-ups and clinic visits, facilitating access to kidney transplantation, and supporting their mental well-being and empowerment to sustain ongoing HD treatment. Despite patients’ demands for health service improvements, the healthcare system in Sri Lanka remains under-resourced to deliver adequate HD services, due to a lack of specialised and trained healthcare professionals, a few state-funded dialysis units, and limited alternative care approaches. To address these timely concerns, there is a need of more pragmatic, person-centred and efficiency-focused interventions that can improve care delivery for these patients.

### Strengths and limitations

This study was a multicenter qualitative study conducted in three main government hospital dialysis units in Sri Lanka and utilised maximum variation sampling, which maintains the transferability of results to patients who are undergoing HD in similar contexts in Sri Lankan hospitals. It provided wider and more thorough insights into the experiences of patients on HD care. This is probably the first multi-centre, qualitative exploration of the experience of HD care among patients in Sri Lankan hospitals. Nevertheless, the study was descriptive in nature and only involved manifest content analysis; therefore, it lacks deep and hidden meanings and interpretations of participants’ experiences.

## Conclusion

This study highlights the complex journey of individuals receiving HD, encapsulated in the overarching theme of ‘Adapting to a Disrupted Life Trajectory’. The findings revealed that living with HD has imposed a substantial burden on daily life and caused a wide range of physical and psychological challenges, indicating the need for more attention and supportive care approaches to improve the health outcomes of patients. Although participants drew strength from their families, peers, and the coping strategies they adapted throughout their journey, they expected their healthcare service would be at a standard level, particularly in terms of quality, safety, and compassion. Therefore, a more holistic, person-centred approach is needed in HD care, one that integrates physical, emotional, social, and spiritual support. Addressing these gaps in service delivery, ensuring compassionate care, and reinforcing multidisciplinary support can significantly improve patients’ ability to adapt and endure treatment-related challenges over time, particularly in low-resource settings where access to HD treatment is inadequate. It is crucial to develop a systematic approach for routine symptom assessment and management, and promote treatment adherence among patients to prevent potential complications associated with disease and HD treatment, particularly through collaboration with a community care team. In the macro level, while looking for avenues to expand HD services, such as in the form of satellite units, the ways to expand alternative treatments, such as home-based PD or HD, and integrate supportive care approaches into routine nephrology care would also be beneficial to address the growing demand for RRT. Future research is key to developing and implementing context-specific, person-centred care interventions for patients undergoing HD in the Sri Lankan hospital context to improve the overall well-being of the patients, their families, thereby reducing the healthcare burden of the country.

## Supplementary Information

Below is the link to the electronic supplementary material.

Supplementary Material 1

Supplementary Material 2

## Data Availability

The datasets used and/or analysed during the current study are available from the corresponding author upon reasonable request.

## Abbreviations

CKD: Chronic Kidney Disease
ACP: Advance Care Planning
