# Supplementary material for: Living with haemodialysis in Sri Lanka: a qualitative study on patient adaptation and care perceptions
Source: BMC Nephrol. 2026 Jan 6;27:22. doi: 10.1186/s12882-025-04728-6 (PMC12784590; doi:10.1186/s12882-025-04728-6)
Supplement: Supplementary file 2 — Supplementary Material 2 [file 12882_2025_4728_MOESM2_ESM.docx]

**Supplementary file 2:** **Interview guide**

**Patient’s feelings and experiences about haemodialysis treatment and care**

1. How do you describe the transition to haemodialysis care from your normal lifestyle?

- How about your behavioural change due to changing diet, fluid restriction, or

having many medications in order to undergo haemodialysis treatment?

2. How do you feel about care related to haemodialysis treatment?

- Tell me about your experience of care in haemodialysis units.

- How does the health care team – doctors, nurses, supportive staff help you when you

attend for haemodialysis treatment?

- How helpful is the health care team while you are on haemodialysis treatment-

physical care, psychological care, vascular access care (care for your cannula site)

- How does the health care team help you after haemodialysis treatment when going

home?

3. How do you perceive your day-to-day life with the care you received?

- Sense of disease burden

- Physical, emotional and psychological impacts

4. Challenges or barriers to receiving proper haemodialysis care

- Did you feel any challenges or barriers related to receiving haemodialysis care in

a proper manner?

**Potential support needs**

5. Can you tell me who supports you with your treatments at your home?

- Involvement of family members, friends or neighbours

- How do they help you with the continuity of treatment?

6. According to your experience, any suggestions to improve care for the patients with CKD

undergoing haemodialysis?
